# No Association between Fish Intake and Depression in over 15,000 Older Adults from Seven Low and Middle Income Countries–The 10/66 Study

**DOI:** 10.1371/journal.pone.0038879

**Published:** 2012-06-19

**Authors:** Emiliano Albanese, Flavia L. Lombardo, Alan D. Dangour, Mariella Guerra, Daisy Acosta, Yueqin Huang, K. S. Jacob, Juan de Jesus Llibre Rodriguez, Aquiles Salas, Claudia Schönborn, Ana Luisa Sosa, Joseph Williams, Martin J. Prince, Cleusa P. Ferri

**Affiliations:** 1 Institute of Psychiatry, King’s College London, London, United Kingdom; 2 Department of Epidemiology, Italian National Institute of Health, Rome Italy; 3 Department of Nutrition and Public Health Intervention Research, London School of Hygiene and Tropical Medicine, London, United Kingdom; 4 Psychogeriatric Unit, National Institute of Mental Health “Honorio Delgado Hideyo Noguchi”, Lima, Perú; 5 Internal Medicine Department, Universidad Nacional Pedro Henriquez Ureña (UNPHU), Santo Domingo, Dominican Republic; 6 Institute of Mental Health, Peking University, Beijing, China; 7 Christian Medical College, Vellore, India; 8 Facultad de Medicina Finley-Albarran, Medical University of Havana, Havana, Cuba; 9 Faculty of Medicine, Universidad Central de Venezuela, Caracas, Venezuela; 10 National Institute of Neurology and Neurosurgery of Mexico, Mexico City, Mexico; 11 Institute of Community Health, Chennai, India; Catholic University of Sacred Heart of Rome, Italy

## Abstract

**Background:**

Evidence on the association between fish consumption and depression is inconsistent and virtually non-existent from low- and middle-income countries. Using a standard protocol, we aim to assess the association of fish consumption and late-life depression in seven low- and middle-income countries.

**Methodology/Findings:**

We used cross-sectional data from the 10/66 cohort study and applied two diagnostic criteria for late-life depression to assess the association between categories of weekly fish consumption and depression according to ICD-10 and the EURO-D depression symptoms scale scores, adjusting for relevant confounders. All-catchment area surveys were carried out in Cuba, Dominican Republic, Venezuela, Peru, Mexico, China, and India, and over 15,000 community-dwelling older adults (65+) were sampled**.** Using Poisson models the adjusted association between categories of fish consumption and ICD-10 depression was positive in India (p for trend = 0.001), inverse in Peru (p = 0.025), and not significant in all other countries. We found a linear inverse association between fish consumption categories and EURO-D scores only in Cuba (p for trend  = 0.039) and China (p<0.001); associations were not significant in all other countries. Between-country heterogeneity was marked for both ICD-10 (I^2^>61%) and EURO-D criteria (I^2^>66%).

**Conclusions:**

The associations of fish consumption with depression in large samples of older adults varied markedly across countries and by depression diagnosis and were explained by socio-demographic and lifestyle variables. Experimental studies in these settings are needed to confirm our findings.

## Introduction

Depression is projected to become the leading cause of the global burden of disease by 2030, [1] with the steepest increases in prevalence in ageing societies [2] and in countries with low and middle incomes. [3], [4] Increased prioritisation of mental health interventions in public health policies is urgently required particularly in these settings [5], [6] where the mental–physical treatment gap is greatest. [7] The prevention and treatment of late-life depression represent a major current research and policy effort, [8] and some protective factors may also have treatment promises.

Lifestyle risk factors including nutrition, vascular pathology and inflammation [9] may influence the heritability of late life depression. [10] The n-3 Long Chain Polyunsaturated Fatty Acids (n-3 LC PUFAs), namely eicosapentaenoic acid (EPA) and docosahexanoic acid (DHA) most commonly found in oily fish play important physiologic roles in humans including their actions on cell signaling and transduction, receptors density regulation and metabolite production.[11]–[13] Plasma n-3 LC PUFA concentrations appear to correlate with serotonin and dopamine status in the central nervous system [14] which may also be important in the pathophysiology of depression. [15], [16].

Ecological studies suggest an inverse association of fish consumption with prevalent depression, [14], [17] post-partum depression [18] and bipolar disorders. [19] Population-based epidemiological studies employing food frequency questionnaires to ascertain diet similarly report inverse associations between fish consumption and prevalent depression,[20]–[22] depressed mood [23] or mental health, [24] although these findings are not consistent, [25], [26] particularly when diet history questionnaires are used. [27], [28] Depression diagnosis in these studies was self-reported using a variety of questionnaires, which may further contribute to the inconsistency in findings. The results of intervention studies which have largely focused on the potential treatment effect of EPA and/or DHA supplementation on depression, have been inconclusive and between study heterogeneity is marked. [29].

Numerous social and demographic factors including gender, marital status, functional impairment and illness influence risk for depression in older people.[30]–[34] The relationships between risk factors are complex and confounding may play an important role in modifying any association of dietary fish consumption with depression. [35] There are currently no studies assessing the association between fish consumption and depression in low and middle income countries and the degree of consistency of the evidence across cultures is virtually unknown.

In the absence of any evidence on the importance of fish consumption as a protective factor against depression in low and middle income countries, we set out to test the strength of the relationship of fish consumption with prevalent depression in large representative samples of community-dwelling older people, aged 65 years and over, in China, India, Cuba, Dominican Republic, Mexico, Peru and Venezuela. We further assess the robustness and consistency of any such associations taking a wide range of potential confounders into account and comparing results obtained using two different diagnostic criteria.

## Materials and Methods

### Ethics Statement

The 10/66 research programme was approved by the Research Ethics Committee at King’s College London and by local ethical committees in each country.

### Study Design

This study is part of the 10/66 research programme on ageing, mental health and non-communicable diseases in countries with low and middle incomes. The study designs and procedures have been extensively described, [36] and validated. [37] Further details are available on the study website www.alz.co.uk/1066. Here we describe in brief the methods and measures directly pertinent to the current report.

Between January 2003 and November 2007, we conducted all catchment area one-phase surveys amongst community dwelling older people (65+ years) in Cuba, Dominican Republic, Peru, Venezuela, Mexico, China and India. We recruited all residents aged 65+ years with no exclusion criteria. Power calculations suggested a target sample size of 2,000 participants per country. Written consents were obtained from all participants or from a close relative or caregiver in case of incapacity or illiteracy.

The defined 10/66 study protocol includes data on household, participants and informants’ socio-demographic characteristics and lifestyle risk factors, the Geriatric Mental State (GMS) examination, [38] physical and neurological examinations (NEUROEX), [39] a comprehensive cognitive battery [40] and a structured informant interview. The study protocol, including the dietary assessment, has been translated, with cultural adaptations, into Spanish, Chinese and Tamil by local physicians fluent in English. A comprehensive study manual (along with videos) covers all procedural and content aspects of the research programme. Local principal investigators received standardized one-week trainings and scrutinized the work of the local teams with the continuous assistance of the London coordinating centre.

### Depression Diagnosis

Depression diagnosis criteria applied in the 10/66 population-based studies have been validated [41] and extensive details on GMS-based criteria and algorithms have been described elsewhere. [42] In this study, with respect to the month preceding the interview, we use the ICD-10 depressive episode criteria, [43] derived using a validated clinical-based computerized algorithm [44] applied to the GMS and disregarding severity. Additionally we use a score derived from the EURO-D [45] scale for late-life depression symptoms, based on 12 GMS domains (depressed mood, pessimism, suicidality, guilt, sleep, interest, irritability, appetite, fatigue, concentration, enjoyment and tearfulness), which ranges from 0 (no symptoms) to 12. Limited to the descriptive analyses we also consider a cut-off point ≥4 to identify cases as in the SHARE studies in Europe. [46] ICD-10 criterion strictly identifies clinically significant cases, while EURO-D diagnosis proved considerably more sensitive in identifying subsyndromal cases whose depressive symptomatology is significantly associated to severe disability. [42] Past history of depression was determined on self-reported clinical diagnosis.

### Dietary Assessments

We asked standardized questions (“how often do you eat fish/meat in a week?”) in face-to-face interviews, and recorded the average weekly consumption (“never”, “some days”, “most days”, “every day”) along with the average number of daily portions of vegetables, fruits and units of alcohol consumed per week. Interviewers gathered confirmations from informants (generally a close relative) when dietary habits appeared implausible (n = 58), or for participants with moderate or severe dementia according to Clinical Dementia Rating scale (CDR) [47] (n = 367). We have previously reported the concurrent validity of our dietary assessment across the 10/66 study sites and tested internal consistency amongst dietary measures (i.e. fish, meat, fruits and vegetables and alcohol) and assessed Kendall’s τ correlations with socio-demographic characteristics. Reported fish consumption followed expected patterns of associations with higher educational levels and better socio-economic circumstances. The dietary intake assessment also identified inverse associations of reported fish consumption with prevalent dementia. [48].

### Other Relevant Measures

We recorded participants’ age (confirmed by documentations and the informants or with respect to historical events) and gender; educational level (in five grades from illiteracy to completed tertiary school); marital status (never married, married/cohabitant, widowed and divorced/separated); number of household assets (car, television, refrigerator, telephone, plumbed toilet, water and electricity utilities) and physical activity level (not at all, fairly active, active and very physically active). We registered self-reported physical impairments and clinically diagnosed illnesses (including stroke, coronary heart disease and diabetes mellitus) using a standard questionnaire. [49] Dementia diagnosis was established applying the cultural and education-fair 10/66 validated algorithm [37], [50] and an overall cognitive score (COGSCORE) was calculated based on our neuropsychological battery. [40].

### Statistical Analysis

#### Participants’ characteristics

We describe the socio-demographic and health characteristics of participants by country and by ICD-10 depression. On inspection we combined participants who reported to eat fish “most days” and “every day”. We then describe weekly fish intakes (“none”, “some days”, “most days”) across countries and by depression status (yes/no) according to ICD-10 criteria and EURO-D caseness. The association of dietary fish intake with participants’ socio-demographic and physical health and dementia characteristics have been reported elsewhere. [48].

#### Association between dietary fish and depression

We used Poisson regressions to calculate prevalence ratios (PRs) with robust 95% confidence intervals, adjusting for clustering of characteristics within households, to determine the risk of prevalent ICD-10 depression associated with level of fish consumption. With the diagnosis of ICD-10 depression as dichotomous outcome we calculated PRs by entering weekly fish intakes in the model as a categorical variable with three levels (‘never eat fish’, ‘eat fish some days’ and ‘eat fish most days’). The middle category of ‘eat fish some days’ was the reference group (PR = 1) in order to aid linearity checks across estimates (i.e. a dose-response-like pattern configures when PR<1 and PR>1 amongst least and most days fish consumers respectively). [51] We then entered fish consumption in the above models as a continuous variable and interpreted results as test for trends and tested departures from linearity applying likelihood ratio tests. We generated an unadjusted and two adjusted models (see below) by country, selecting confounders on the basis of our a priori hypothesis and consistently with previous population-based studies to allow comparisons. In adjusted model 1 we controlled for age (continuous variable), gender (females vs. males), educational level (none, some, primary, secondary and tertiary), number of household assets (continuous variable), marital status (never married, married/cohabiting, widowed and divorced/separated), overall cognitive score (continuous variable), self-reported clinically diagnosed diabetes, coronary heart disease and stroke (yes/no) and total number of physical illnesses (continuous variable). To disaggregate the possible effect of socio-demographic from lifestyle factors, adjusted model 2 further allowed for physical activity level (very, fairly, not very, not at all physically active), weekly meat intake (never, some days, most days, every day), fruits and vegetables weekly portions consumed (continuous variable) and units of alcohol drunk (continuous variable). The country-specific differential contribution of confounders was explored performing backward stepwise estimations. We combined the country-specific pairs of PRs calculated in the crude model and in model 1 and 2 into a series of fixed-effect method meta-analyses to obtain the pooled estimates of the associations between fish intake levels and ICD-10 depression status, after having formally tested heterogeneity with Cochrane Q statistics (on appropriate degrees of freedom) and calculated I^2^ Higgins to determine the percentage of between country differences not due to chance. [52].

On inspection EURO-D scores were skewed and over-dispersed with an apparent zero-inflation. To investigate this zero-inflation, we modeled the effect of country on EURO-D scores using zero-inflated negative binomial (ZINB) regression. With this method the assumption is made that a subgroup of participants exists who always has zero counts regardless (the so called ‘nay-saying’). In zero-inflated models, this group is referred to as the ‘certain zeros’. We calculated the likelihood of being a ‘certain zero’ by country with a logit specification and used negative binomial models in the non-certain zero group to determine the effect on the EURO-D scores of country alone (dummy variable) and then adjusting for compositional variables: age, gender, education, household assets, self-reported diabetes, coronary-heart disease and stroke, number of physical illnesses, marital status, overall cognitive score and physical activity level. In doing so, we sought to account for the different psychometric properties of the EURO-D scale across the study sites.

Next we measured the associations between fish intake and depressive symptomatology entering dietary fish categories (independent variable) and EURO-D scores (dependent/outcome variable) in ZINB models, similarly to other population-based studies that used comparable depression scale and likewise had to deal with excessive zero values. [53] We used Vuong tests to formally test the goodness of fit of the ZINB over standard negative binomial models. [54] We obtained relative risks (RR) by country first in unadjusted models and then controlling for confounders as in model 1 and 2 (see above) and we estimated degree of heterogeneity among site-specific estimates using Higgins I^2^ with 95% C.I. as appropriate.

## Results

The achieved sample was 15,022, response rates were over 80% in all countries. Data on depression diagnosis and fish consumption were available for 14,926 participants (99.4% of the total). Some socio-demographic differences between countries especially in age, education, co-habitation and asset ownership were identified, and differences between those with and without depression were marked (Table 1). ICD-10 depression prevalence ranged from 0.5% in China to 13.8% in Dominican Republic, and EURO-D depression prevalence was consistently higher ranging from 2.8% in China to 41.5% in India. Self-reported clinically diagnosed stroke prevalence varied from 1.6% in India to 8.7% in the Dominican Republic, while self-reported non-insulin dependent diabetes mellitus was particularly common in Mexico and Cuba (21.7% and 18.6% respectively). Overall in Venezuela and the Dominican Republic more people suffered from three or more physical illnesses than in the other countries. As previously reported 10/66 dementia rate varied from 6.3% to 11.7% between countries. [55].

**Table 1:** Participants Socio-Demographic and Health Characteristics.

Variable	Cuba	Dominican Republic	Peru	Venezuela	Mexico	China	India	Total ICD-10depressiveepisode non-cases	Total ICD-10depressiveepisode cases	P value
**Response rate** (%)	94.0	95.0	84.0	80.0	85.0	85.0	85.0	-	-	
**Achieved sample** (n)	2944	2011	1933	1965	2003	2162	2004	14123	899	
**Age** (missing values)	7	0	1	4	1	0	4	17	1	
65–69 (%)	25.9	26.5	28.7	42.8	27.2	32.3	37.3	31.5	26.5	<0.001
70–74 (%)	26.9	25.9	25.5	23.9	29.0	30.4	33.4	27.9	26.8	
75–79 (%)	21.7	19.7	20.6	17.6	21.3	21.1	16.1	19.8	21.2	
80 and over (%)	25.5	27.9	25.2	15.7	22.5	16.1	13.2	20.8	25.5	
**Gender** (missing values)	0	2	0	33	0	0	15	50	0	
Female (%)	65.0	65.9	61.2	62.4	63.3	56.3	55.7	61.6	70.5	<0.001
**Education** (missing values)	8	19	16	40	0	0	2	72	13	
No education (%)	2.6	19.7	6.3	8.1	27.7	37.5	54.4	21.0	28.6	<0.001
Some Education (%)	22.3	51.3	12.1	23.1	43.1	12.4	21.4	25.6	36.0	
Complete Primary School (%)	33.3	18.6	37.9	50.1	17.5	26.0	16.4	29.1	22.0	
Complete Secondary School (%)	24.8	6.8	27.0	13.8	6.2	17.6	5.6	15.6	8.1	
Complete Tertiary School (%)	17.0	3.7	16.7	4.8	5.5	6.6	2.2	8.8	5.3	
**Marital status** (missing values)	8	15	11	45	1	0	3	72	11	
Never married (%)	9.4	7.0	11.1	9.8	5.2	1.2	1.3	6.5	5.7	<0.001
Married/cohabiting (%)	43.3	29.4	56.8	48.0	50.4	65.4	50.2	49.7	34.8	
Widowed (%)	31.6	40.4	27.3	28.6	38.3	33.3	46.1	34.3	44.1	
Divorced/separated (%)	15.7	23.3	4.8	13.6	6.1	0.1	2.4	9.4	15.3	
**Number of assets** (missing values)	8	5	0	0	0	1	4	18	0	
Three or less (%)	2.7	15.2	4.9	2.0	21.6	5.2	52.4	13.5	22.9	<0.001
**Dementia** (missing values)	13	0	2	1	0	0	0	16	0	
Meets criteria for 10/66 dementia (%)	10.7	11.7	8.5	7.1	8.5	6.3	9.0	8.4	18.5	<0.001
**Depression** (missing values)	0	0	0	0	0	0	0	0	0	
Any ICD-10 depressive episode (%)	4.9	13.8	5.3	5.5	4.6	0.5	8.2	n/a	n/a	
EURO-D (%)	23.6	38.0	28.4	29.5	28.7	2.8	41.5	22.4	98.0	<0.001
**Self-reported diagnosed NCDs** (missing values)	7	2	5	33	0	0	1	39	9	
Stroke (%)	7.8	8.7	6.9	7.0	7.0	5.9	1.6	6.2	11.7	<0.001
Coronary heart disease (%)	8.1	4.6	3.5	9.6	3.1	16.6	1.2	6.6	10.8	<0.001
Diabetes mellitus (%)	18.6	14.0	9.0	16.0	21.7	9.4	9.3	13.9	19.1	<0.001
**Number of physical illnesses** (missing values)	6	2	2	33	0	0	1	35	9	
Three or more physical illnesses (%)	9.9	23.1	13.7	25.3	17.1	11.4	10.4	13.8	40.8	<0.001

Abbreviations: ICD-10 = International Classification of Diseases (10^th^ edition); EURO-D = EURODEP Concerted Action Programme common depression symptoms scale NCDs = Non-communicable diseases.

In all countries, most participants reported fish consumption on some days of the week (as shown in table 2). In all countries except India those reporting the consumption of fish on most days of the week had lower proportions of ICD-10 depression and lower EURO-D scores compared to those reporting to never eat fish. Moreover, most of the variation in the distribution of EURO-D scores across countries was accounted for by China and to a lesser extent Cuba. The very high proportion of zero scores (zero-inflation) (81.1%) in China lowered the country mean score (Table 2).

**Table 2:** ICD-10 depression cases and EURO-D scores (and number of zero scores) by fish consumption categories.

	Depression status		Weekly Fish Intake		
Country			Never	Some days	Most days
**Cuba**	ICD-10 depression	No n (%)	270 (94.1)	2229 (94.9)	291 (97.3)
		Yes n (%)	17 (5.9)	119 (5.1)	8 (2.7)
	EURO-D depression	Mean score (sd) [n]	2.6 (2.5) [279]	2.1 (2.4) [2312]	1.6 (1.9) [291]
		Zero scores n (%)	76 (27.2)	778 (33.7)	119 (40.9)
		Mean score omitting zeros (sd) [n]	3.6 (2.3) [203]	3.2 (2.2) [1534]	2.7 (1.8) [172]
**Dominican Republic**	ICD-10 depression	No n (%)	588 (86.0)	998 (86.2)	136 (86.6)
		Yes n (%)	96 (14.0)	160 (13.8)	21 (13.4)
	EURO-D depression	Mean score (sd) [n]	3.3 (2.6) [678]	2.9 (2.6) [1145]	2.6 (2.5) [157]
		Zero scores (%)	105 (15.5)	241 (21.1)	42 (26.8)
		Mean score omitting zeros (sd) [n]	4.0 (2.3) [573]	3.7 (2.4) [904]	3.5 (2.3) [115]
**Peru**	ICD-10 depression	No n (%)	148 (91.9)	1337 (94.6)	340 (96.3)
		Yes n (%)	13 (8.1)	76 (5.4)	13 (3.7)
	EURO-D depression	Mean score (sd) [n]	2.7 (2.5) [150]	2.5 (2.2) [1382]	2.6 (2.1) [346]
		Zero scores (%)	36 (24)	283 (20.5)	68 (19.7)
		Mean score omitting zeros (sd) [n]	3.5 (2.3) [114]	3.2 (2.1) [1099]	3.2 (1.8) [278]
**Venezuela**	ICD-10 depression	No n (%)	82 (92.1)	801 (93.4)	928 (96.5)
		Yes n (%)	7 (7.9)	57 (6.6)	34 (3.5)
	EURO-D depression	Mean score (sd) [n]	2.8 (2.3) [88]	2.6 (2.4) [854]	2.32 (2.2) [956]
		Zero scores (%)	18 (20.5)	204 (23.9)	255 (26.7)
		Mean score omitting zeros (sd) [n]	3.6 (1.9) [70]	3.4 (2.2) [650]	3.2 (2.0) [701]
**Mexico**	ICD-10 depression	No n (%)	534 (94.2)	1274 (95.9)	97 (95.1)
		Yes n (%)	33 (5.8)	54 (4.1)	5 (4.9)
	EURO-D depression	Mean score (sd) [n]	2.6 (2.2) [560]	2.4 (2.3) [1315]	2.3 (2.2) [102]
		Zero scores (%)	119 (21.3)	317 (24.1)	27 (26.5)
		Mean score omitting zeros (sd) [n]	3.3 (2.0) [441]	3.2 (2.1) [998]	3.1 (2.0) [75]
**China**	ICD-10 depression	No n (%)	66 (98.5)	1461 (99.6)	625 (99.5)
		Yes n (%)	1 (1.5)	6 (0.4)	3 (0.5)
	EURO-D depression	Mean score (sd) [n]	1.2 (1.8) [59]	0.4 (1.1) [1425]	0.2 (0.9) [616]
		Zero scores (%)	34 (57.6)	1114 (78.2)	555 (90.1)
		Mean score omitting zeros (sd) [n]	2.8 (1.8) [25]	1.9 (1.6) [311]	2.3 (2.0) [61]
**India**	ICD-10 depression	No n (%)	395 (93.6)	1298 (91.2)	140 (92.1)
		Yes n (%)	27 (6.4)	126 (8.8)	12 (7.9)
	EURO-D depression	Mean score (sd) [n]	2.7 (2.8) [407]	3.3 (2.8) [1389]	3.9 (2.7) [152]
		Zero scores (%)	107 (26.3)	274 (19.7)	20 (13.2)
		Mean score omitting zeros (sd) [n]	3.7 (2.6) [300]	4.1 (2.6) [1115]	4.5 (2.4) [132]

Abbreviations: ICD-10 = International Classification of Diseases (10^th^ edition); EURO-D =  EURODEP Concerted Action Programme common depression. Sd =  standard deviation.

The first ZINB model assessed the effect of country on EURO-D scores and showed that most of the variation arose from zero-inflation while the variation was markedly smaller in the count part of the model. After adjustment, for both models the effect of the compositional differences between countries in socio-demographic and health characteristics was modest (Table 3).

**Table 3:** Between-country variation in zero inflation^1^ and euro-d total score counts as modelled by zero-inflated negative binomial regression, before and after adjustment for compositional factors.

	Crude model	Adjusted model*
**Zero Inflation**		
Cuba	1 (reference)	1 (reference)
DR	0.49 (0.39, 0.62)	0.33 (0.24, 0.45)
Peru	0.30 (0.23, 0.38)	0.24 (0.17, 0.34)
Venezuela	0.50 (0.38, 0.65)	0.38 (0.27, 0.54)
Mexico	0.51 (0.40, 0.65)	0.40 (0.30, 0.54)
China	13.47 (10.74, 16.88)	10.66 (8.47, 13.43)
India	0.43 (0.33, 0.56)	0.42 (0.32, 0.55)
**Count**		
Cuba	1 (reference)	1 (reference)
DR	1.25 (1.19, 1.32)	1.05 (0.99, 1.11)
Peru	1.03 (0.97, 1.09)	1.07 (1.01, 1.14)
Venezuela	1.03 (0.96, 1.10)	0.88 (0.83, 0.95)
Mexico	1.03 (0.97, 1.09)	0.93 (0.88, 0.98)
China	0.49 (0.44, 0.55)	0.43 (0.39, 0.49)
India	1.36 (1.29, 1.44)	1.30 (1.22, 1.39)

1 Data for zero inflation are odds ratios (95% confidence interval); data for count model are ratio of counts (95% confidence interval).

* Adjusted for age, gender, educational level, number of household assets, self-reported stroke, diabetes, number of physical illnesses, marital status, overall cognitive score and physical activity level.

### Association between Prevalence of Depression and Dietary Fish

In unadjusted analysis, compared to those who ate fish some days of the week, there was a general tendency for rates of prevalent depression to be higher among those who reported never eating fish and lower among those who reported eating fish most days of the week (Table 4). India and Mexico were exceptions to this general pattern and for ICD-10 criteria there were too few cases in China to estimate parameters. Similarly, EURO-D scores were highest amongst those who reported never eating fish and the general pattern was consistent between ICD-10 diagnosis and EURO-D scores in all countries except Peru. The observed tendency of an inverse association of fish consumption with ICD-10 depression prevalence and EURO-D scores was largely attenuated when adjusted for participants’ socio-demographic and health status (model 1) and lifestyle characteristics (model 2). Stepwise estimations of the covariates coefficients included in model 2 failed to identify a common pattern among countries.

**Table 4:** Crude and adjusted robust prevalence ratios (PRs) (with 95% confidence intervals, CI) from Poisson regressions for the association between ICD-10 depression and fish consumption by country and pooled estimates from fixed-effect models meta-analyses.

	Sample size	Dietary Fish	Prevalence ratio (95%CI)					
Country			Unadjusted	P value*	Model 1^1^	P value*	Model 2^2^	P value*
Cuba	2864	*Never eat fish*	1.19 (0.73 to 1.94)		1.05 (0.64 to 1.72)		1.00 (0.60 to 1.65)	
		*Eat fish some days*	1 (reference)	0.048	1 (reference)	0.073	1 (reference)	0.299
		*Eat fish most days*	0.54 (0.27 to 1.10)		0.51 (0.25 to 1.02)		0.61 (0.31 to 1.22)	
Dominican Republic	1940	*Never eat fish*	0.99 (0.78 to 1.25)		0.88 (0.70 to 1.11)		0.89 (0.71 to 1.12)	
		*Eat fish some days*	1 (reference)	0.981	1 (reference)	0.080	1 (reference)	0.065
		*Eat fish most days*	0.97 (0.64 to 1.49)		1.32 (0.88 to 1.99)		1.4 (0.94 to 2.1)	
Peru	1841	*Never eat fish*	1.62 (0.92 to 2.84)		1.48 (0.84 to 2.62)		1.67 (0.98 to 2.87)	
		*Eat fish some days*	1 (reference)	0.017	1 (reference)	0.065	1 (reference)	0.025
		*Eat fish most days*	0.64 (0.36 to 1.15)		0.72 (0.40 to 1.30)		0.70 (0.38 to 1.27)	
Venezuela	1124	*Never eat fish*	0.70 (0.17 to 2.87)		0.40 (0.07 to 2.35)		0.43 (0.07 to 2.67)	
		*Eat fish some days*	1 (reference)	0.118	1 (reference)	0.162	1 (reference)	0.310
		*Eat fish most days*	0.60 (0.34 to 1.05)		0.51 (0.30 to 0.87)		0.57 (0.32 to 1.03)	
Mexico	1954	Never eat fish	1.53 (0.98 to 2.38)		1.29 (0.79 to 2.11)		1.25 (0.78 to 2.00)	
		*Eat fish some days*	1 (reference)	0.155	1 (reference)	0.477	1 (reference)	0.614
		*Eat fish most days*	1.25 (0.51 to 3.08)		1.23 (0.51 to 2.96)		1.36 (0.58 to 3.21)	
China	2156	Never eat fish	†		†		†	
		*Eat fish some days*	1 (reference)		1 (reference)	n/a	1 (reference)	n/a
		*Eat fish most days*	†		†		†	
India	1868	Never eat fish	0.66 (0.44 to 1.01)		0.71 (0.47 to 1.07)		0.51 (0.32 to 0.83)	
		*Eat fish some days*	1 (reference)	0.133	1 (reference)	0.188	1 (reference)	0.001
		*Eat fish most days*	0.85 (0.48 to 1.54)		0.91 (0.50 to 1.65)		2.47 (1.34 to 4.55)	
**Meta-analysis**	13747							
Pooled estimate		*Never eat fish*	1.07 (0.91 to 1.25)		0.95 (0.81 to 1.12)		0.93‡ (0.78 to 1.10)	
Cochrane Q (degrees of freedom)			Q = 7.15 (5) p = 0.21		Q = 4.23 (5) p = 0.52		Q = 13.09 (5) p = 0.02	
I^2^ Higgins (95% CI)			30% (0 to 71)		0% (0 to 75)		62% (7 to 84)	
		*Eat fish some days*	1 (reference)	<0.001	1 (reference)	<0.001	1 (reference)	0.008
Pooled estimate		*Eat fish most days*	0.73 (0.59 to 0.91)		0.83 (0.67 to 1.03)		1.07 (0.85 to 1.36)	
Cochrane Q (degrees of freedom)			Q = 6.52 (5) p = 0.26		Q = 12.15 (5) p = 0.03		Q = 18.16 (5) p = 0.003	
I^2^ Higgins (95% CI)			23% (0 to 67)		59% (0 to 83)		72% (37 to 88)	

1 Adjusted for: age, gender, educational level, number of household assets, marital status, self-reported diagnosed diabetes, coronary heart disease and stroke, number of physical illnesses, and overall cognitive status.

2 As for model 1 plus weekly meat intake, fruits and vegetables consumption, alcohol intake and physical activity level.

* Test for trend.

† Too few cases to estimate parameters.

‡ Pooled estimates for model 2 are presented to allow direct comparisons with model 1 and un-adjusted models but should be interpreted with caution due to the markedly high between-country heterogeneity.

Fixed-effect meta-analytical combinations of country-specific PRs (and 95% CI) substantially confirmed the patterns observed at the individual country level. In the unadjusted analysis, compared to those who eat fish some days of the week there was a significant decreased risk of prevalent ICD-10 depression among those who eat fish on most days (pooled PR = 0.73; 95% CI: 0.59 to 0.91). Adjusting for socio-demographic, health and lifestyle characteristics markedly attenuated all associations in the pooled analyses (model 2). Across countries a trend emerged towards an increased risk of depressive symptoms (EURO-D criteria only) among those who eat fish on most days compare to those who eat fish some days (Table 5). However, while between-country heterogeneity was moderate and not significant for ICD-10 criteria, it was marked for EURO-D scores and consistently increased from less to more heterogeneity for the adjusted models, such that meta-analysis of the latter estimates were deemed inappropriate (Table 5).

**Table 5:** Relative risks (95% confidence intervals, CI) for the association between euro-D total score and fish consumption from zero-inflated negative binomial (ZINB) models and between country heterogeneity estimates.

	Sample size			Dietary Fish		Relative risks (95%CI)					
Country						Unadjusted	P value*	Model 1^1^	P value*	Model 2^2^	P value*
Cuba	2810		*Never eat fish*			1.17 (1.03, 1.32)		1.12 (0.99, 1.26)		1.12 (0.98, 1.27)	
			*Eat fish some days*			1 (reference)	0.072	1 (reference)	0.003	1 (reference)	0.039
			*Eat fish most days*			0.79 (0.68, 0.90)		0.74 (0.65, 0.85)		0.80 (0.69, 0.91)	
Dominican Republic	1909		*Never eat fish*			1.09 (1.01, 1.18)		1.07 (0.99, 1.15)		1.06 (0.99, 1.14)	
			*Eat fish some days*			1 (reference)	0.275	1 (reference)	0.149	1 (reference)	0.223
			*Eat fish most days*			0.94 (0.82, 1.09)		1.02 (0.89, 1.16)		1.03 (0.90, 1.17)	
Peru	1789		Never eat fish			1.14 (0.99, 1.31)		1.11 (0.97, 1.27)		1.13 (0.99, 1.30)	
			*Eat fish some days*			1 (reference)	0.952	1 (reference)	0.385	1 (reference)	0.494
			*Eat fish most days*			0.98 (0.89, 1.08)		1.03 (0.94, 1.13)		1.01 (0.92, 1.11)	
Venezuela	1101		Never eat fish			0.98 (0.76, 1.25)		0.93 (0.74, 1.17)		0.96 (0.76, 1.21)	
			*Eat fish some days*			1 (reference)	0.057	1 (reference)	0.011	1 (reference)	0.077
			*Eat fish most days*			0.91 (0.81, 1.00)		0.88 (0.80, 0.98)		0.91 (0.83, 1.01)	
Mexico	1935		Never eat fish			1.02 (0.93, 1.10)		0.97 (0.89, 1.06)		0.96 (0.88, 1.05)	
			*Eat fish some days*			1 (reference)	0.803	1 (reference)	0.469	1 (reference)	0.312
			*Eat fish most days*			0.93 (0.77, 1.11)		0.95 (0.80, 1.13)		0.95 (0.80, 1.13)	
China	2089		Never eat fish			2.05 (1.29, 3.25)		1.66 (1.02, 2.70)		1.83 (1.11, 3.01)	
			*Eat fish some days*			1 (reference)	0.393	1 (reference)	0.007	1 (reference)	<0.001
			*Eat fish most days*			0.78 (0.58, 1.04)		0.62 (0.47, 0.83)		0.49 (0.35, 0.67)	
India	1818		Never eat fish			0.84 (0.76, 0.93)		0.86 (0.78, 0.95)		0.86 (0.76, 0.96)	
			*Eat fish some days*			1 (reference)	0.984	1 (reference)	0.578	1 (reference)	0.099
			*Eat fish most days*			1.14 (0.99, 1.31)		1.17 (1.03, 1.33)		1.35 (1.16, 1.58)	
**Between-country heterogeneity**											
I^2^ Higgins (95% CI)			*Never eat fish*			83% (75, 93)		71% (36, 87)		66% (23, 85)	
			*Eat fish some days*			•		•		•	
I^2^ Higgins (95% CI)			*Eat fish most days*			75% (47, 88)		78% (54, 89)		69% (0, 79)	

NOTE: All Vuong test for ZINB vs. standard negative binomial were statistically significant (p<0.001).

1 Adjusted for: age, gender, educational level, number of household assets, marital status, self-reported diagnosed diabetes, coronary-heart disease and stroke, number of physical illnesses, and overall cognitive status.

2 As for model 1 plus weekly meat intake, fruits and vegetables consumption, alcohol intake and physical activity level.

* Test for trend.

## Discussion

We conducted catchment area surveys of representative samples of 15,022 older people in Cuba, Dominican Republic, Venezuela, Mexico, Peru, India and China. We achieved high response rates and applied identical standardized validated protocols in each study site. [50] We used diagnoses of depression cross-culturally validated in older people and face-to-face dietary assessments. [41] Our assessments were well tolerated and confirmed by proxy informants (generally a close relative) where necessary. We identified differences among the seven countries in socio-demographic and health characteristics, and exposure and outcome status. We applied a strict diagnostic criteria of depressive illness (ICD-10) and scores of a broader criterion (EURO-D) to capture depressive symptomatology of less severe or precursor “depressed mood” cases. Overall we found that the associations of high fish consumption with ICD-10 prevalent depression and of low fish consumption with EURO-D scores for depressive symptoms were almost entirely explained by socio-demographic, lifestyle and health characteristics.

While the country-specific estimates of the associations of fish consumption with prevalent depression were overall homogeneous across the Latin American countries, results from India were markedly different. From cross-sectional studies such as this it is not possible to define causality and the unexpected finding in India warrants further research. For example we have recently reported an increased risk of mortality among depressed individuals in our Indian study site, [56] which opens the possibility that survival bias may have modified our associations; namely shorter survival among depressed participants who eat less fish may have occurred such that the high fish consumption in prevalent cases appeared spuriously high. Alternatively, we cannot exclude that depression may lead to an increase of fish consumption in India, differently from all other countries. The paucity of cases of depression in China did not allow appropriate comparisons, and also warrants exploration when data from the incidence phase of the 10/66 project will be available.

The study has some limitations which mainly relate to the study design and dietary assessment. Results based on an all-catchment area sampling procedure should be generalised with caution and only to populations similar to the ones under study. Observational studies hint at but cannot prove causality and are prone to residual confounding. Depression status may influence diet and lifestyle and reverse causality cannot be excluded in cross-sectional. [57].

While there is biological plausibility to support the relationship between fish consumption and depression, [58] the reliability of reported fish consumption as a proxy of n-3 LC PUFAS intake [59] or status [60] has been questioned. Our dietary assessments also provided no information about type of fish consumed or cooking methods which may further modify n-3 LC PUFAS intake. Formal validation of dietary measures in large epidemiological studies is complex especially in multi-country studies such as ours. We validated our dietary assessment using concurrent measures gathered within our study and showed consistent and plausible dietary patterns and important associations with health outcomes. [48] A recent exercise to create detailed lists of common fish and fish names in each study site confirmed that the definition of fish given in our study protocol remains valid across countries.

Information bias is less likely in face-to-face interviews used in our study than in widely used food frequency questionnaires, [61] and systematic underreporting found for example in obese subjects, [62] is also unlikely in our study. Errors in measures of dietary consumption are likely to have occurred at random in our study leading to an underestimation of the true effect. In our study, fish consumption was fairly consistently associated with higher education and better socio-economic conditions, [48] and when we included these potential confounding factors in our models the association of fish consumption with depression was significantly attenuated in all study sites.

Our depression diagnoses are based on symptoms referred to the month prior the interview and we did not distinguish between long life depression and late life depression. However, a sensitivity analysis that excluded those who reported a past history of clinically diagnosed depression did not alter substantially our results (data not shown). Our diagnostic tools have been cross-culturally validated [41] and the internal validity of our study is strong, nevertheless the large between-country variation in zero inflation in the EURO-D scores (independent of compositional factors) represents an important finding, with a large positive association with zero inflation in China and inverse associations in all other countries interpreted as a cultural tendency to so-called nay-saying in China and yea-saying in the other settings compared to our reference country Cuba (where access to health care is universal and number of psychiatrists per inhabitant highest). Beyond this, between countries comparisons are appropriate and indeed the low prevalence of depression in China compared to figures from several European countries [63] deserves further investigation. For instance it may be that by favouring a more “etic” vs. a less “emic” approach, we might have involuntarily introduced a cultural bias. Finally, complete data for all the covariates were available for 90.5% of the whole sample. Those with and without missing information did not differ for depression prevalence (p = 0.258) and fish consumption patterns (p = 0.09) and we ran all analyses on the same smallest sample sizes by country allowing direct comparisons across models. However while generally robust, some of our estimates from Venezuela should be interpreted conservatively due to high missing data on alcohol consumption (n = 803). When we repeated the analyses including these participants (and excluding alcohol consumption from model 2) 95% C.I. were smaller and estimates similar, for example the PRs of ICD-10 depression for those who never eat fish compared to those who eat fish some days were 1.17 (95% CI: 0.52, 2.65), 0.97 (95%CI: 0.43, 2.19) and 0.52 (95%CI: 0.34, 2.78) for the unadjusted model and model 1 and 2 respectively.

To our knowledge this is the first population-based study to focus exclusively on late-life depression and fish consumption, and the largest study ever reported in which depressive status was ascertained by applying two validated diagnostic criteria. Importantly, this is also the first study that compared data collected using a standardised protocol from a set of culturally and geographically diverse low and middle income countries. While our results differ from some previous reports[20]–[24], [64], [65] they replicate the findings from two similar large studies of participants aged 50+ years in Finland. [27], [66] Moreover, our results are broadly consistent with studies that defined more than two categories of fish consumption [25], [26], [28], [67] and that adjusted for a similar range of potential confounding variables. [25], [35].

Inconsistency of results between observational studies may be due to differences in methodology, populations studied and diagnostic procedures, but it could indeed reflect a genuine absence of an association of dietary fish consumption with depressive status. The latter interpretation is consistent with null experimental findings on the effect of omega-3 supplementation to improve depressive symptoms in populations that are not deficient [68] and is in line with the findings of a recent updated systematic review of similar randomized controlled trials. [29] It has been hypothesized that gene polymorphisms, [69] that alter n-3 LC PUFAS absorption and metabolism, may explain some of this inconsistency, [70], [71] but the multi-factorial nature of depression suggests that genes may in fact interact with lifestyle factors and the environment in a very complex manner, which may over-shadow the role of fish and n-3 LC PUFAS.

Our results for the first time extend to low and middle income countries the general finding from large population-based and intervention studies that there is no evidence of an association of fish consumption with depression in later life. Experimental studies are surely needed to clarify any underlying biochemical and physiological mechanisms with which n-3 LC PUFAS may interact with mood disorders and depression. However, while fish represents a healthy dietary choice,[72]–[74] our results do not support the recommendation to increase fish consumption to prevent late life depression among older people in countries with low and middle incomes. Other modifiable risk factors should be targeted and prioritized and other actions taken [6] to address the forecast global epidemic of depression.
